# Diagnostic Value of Thunderclap Headache and Convexal Subarachnoid Hemorrhage for Reversible Cerebral Vasoconstriction Syndrome: A Case Report

**DOI:** 10.7759/cureus.20411

**Published:** 2021-12-14

**Authors:** Ricardo D Otiniano-Sifuentes, Laura Zelada-Ríos, Jorge Ramírez-Quiñones, Carlos Abanto, María Novoa, Pilar Calle La Rosa, Néstor Flores, Lourdes Simbrón-Ribbeck, Ana Valencia, Danny Barrientos-Imán

**Affiliations:** 1 Departamento de Enfermedades Neurovasculares, Instituto Nacional de Ciencias Neurológicas, Lima, PER; 2 Departamento de Diagnóstico por Imágenes, Instituto Nacional de Ciencias Neurológicas, Lima, PER; 3 Departamento de Enfermedades Neurovasculares, Instituto Nacional de Ciencias Neurológica, Lima, PER

**Keywords:** stenosis, intracranial vasoconstriction, thunderclap headache, convexal subarachnoid hemorrhage, reversible cerebral vasoconstriction syndrome

## Abstract

Reversible cerebral vasoconstriction syndrome (RCVS) is an underdiagnosed cause of convexal subarachnoid hemorrhage, characterized by thunderclap headache associated with focal and segmental intracranial vasoconstriction. It can appear complications such as intracerebral hemorrhage, seizures, posterior reversible leukoencephalopathy, or ischemic stroke. Our objective is to present the case of a 51-year-old woman with an RCVS diagnosis, who had a normal digital subtraction angiography at the illness onset. We highlight the high diagnostic value of thunderclap headache and convexal subarachnoid hemorrhage. We also highlight the importance of repeating the angiographic studies in the second week when there is strong diagnostic suspicion.

## Introduction

Reversible cerebral vasoconstriction syndrome (RCVS) is a unified term proposed to describe an entity characterized by thunderclap headache associated with focal segmental vasoconstriction that resolves spontaneously within three months [1]. However, angiographic studies can be normal in the first week of illness and erroneously rule out the diagnosis of RCVS [2]. We present the case of a young woman with normal digital subtraction angiography (DSA) at the onset of her disease, but the characteristics of the thunderclap headache and the early finding of convexal subarachnoid hemorrhage (cSAH) allowed us to reaffirm the possibility of RCVS.

## Case presentation

A 51-year-old female with no significant medical history, who seven days before admission presented with a mild headache of recent onset. After that, two days before her admission, while she was exercising, she had a sudden, predominantly frontal, throbbing, holocranial headache, which in a matter of seconds reached an intensity of 9/10, associated with vomiting. The headache partially subsided after ingestion of analgesics. During the next two days, she had two similar episodes of headache associated with vomiting, after defecation. Due to the persistence of the condition, she came to the emergency department of this institution.

**Figure 1:**
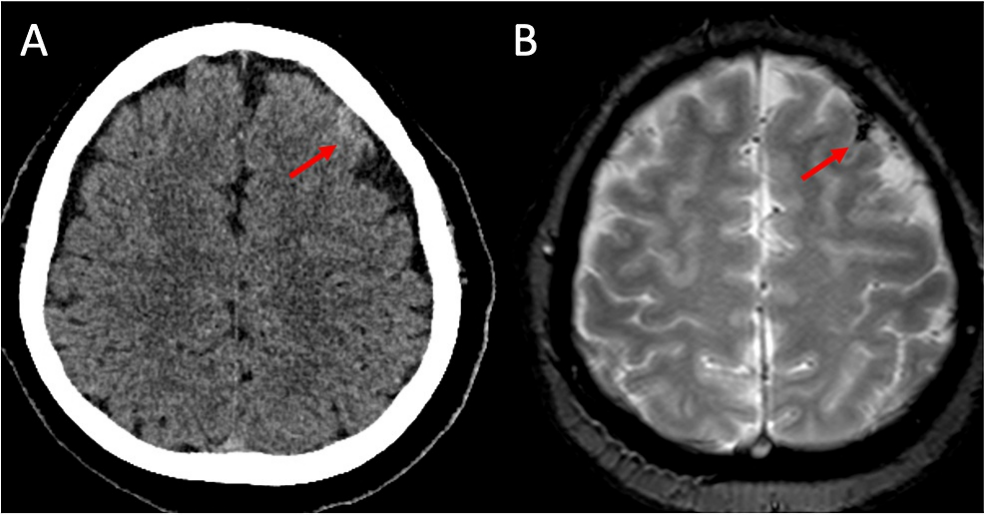
Convexal subarachnoid hemorrhage Brain non-contrast computed tomography at seven years of disease showed scarce hyperdense blood content in the upper left frontal sulcus (A), which correlates with hemosiderin deposition in the T2-weighted gradient-echo MRI (B).

**Figure 2:**
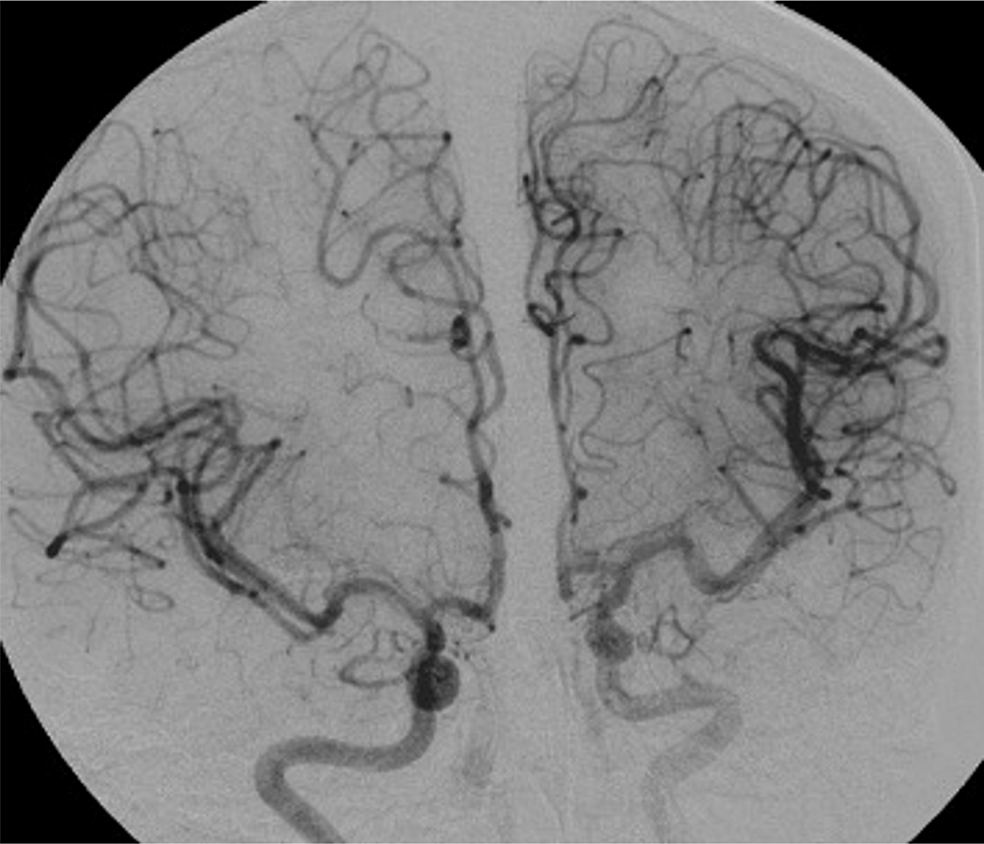
Digital subtraction angiography (DSA) carried out at seven disease days showing no aneurysmal dilatations or alterations in vascular tone

On neurological examination, the patient was oriented and alert, with a painful face, isochoric and photoreactive pupils, and did not present neurological focalization or meningeal signs. In the cerebrospinal fluid study, protein and cell values were normal and there was no glucose consumption. On admission brain non-contrast computed tomography, hyperdensity was evidenced in the left prefrontal convex grooves, which is why it was classified as subarachnoid hemorrhage (Figure 1A). However, the DSA study performed at seven days of illness onset did not reveal alterations (Figure 2). The magnetic resonance imaging (MRI) showed hypointensity in the T2-weighted gradient-echo and magnetic susceptibility sequences at the level of the subarachnoid space of the convexity of the left superior frontal sulcus, confirming the diagnosis of cSAH (Figure 1B).

**Figure 3:**
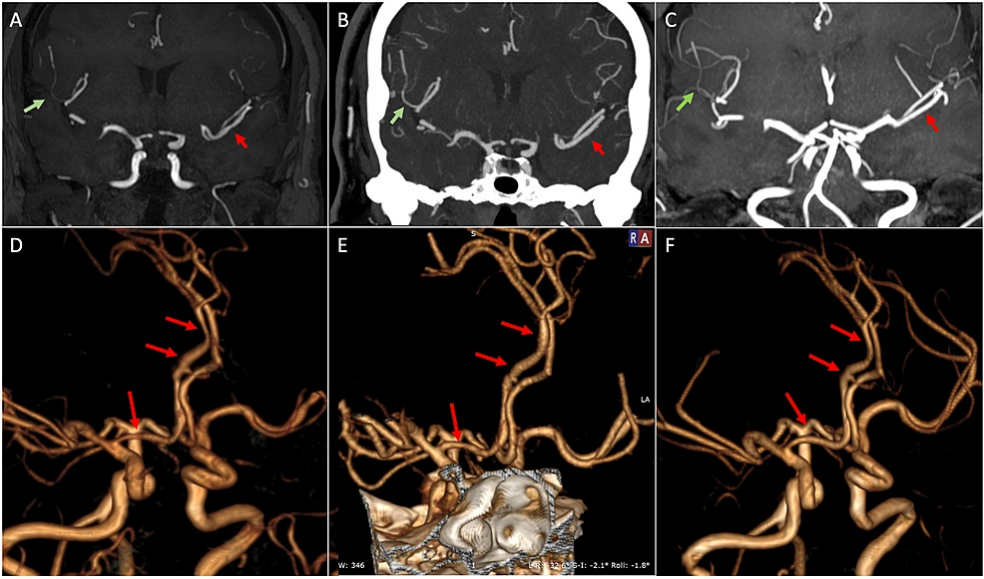
Segmental stenosis of intracranial branches (A, D): TOF angiography performed at 15 days of illness shows irregularity due to segmental stenosis of the ventral branch of the left MCA (red arrow) and of a right M4 cortical artery (green arrow). Irregularities with areas of stenosis and dilations of the right anterior cerebral artery are also observed. (B, E): In the CT angiography performed 21 days after the onset of the disease, there is complete resolution of the stenosis in the ventral branch of the left MCA and partial resolution in the right cortical artery. In the TOF angiography performed at three months (C, F), there were no findings of vasoconstriction.

The magnetic resonance angiography (MRA) was performed after 15 days of illness onset, showing segmental stenosis of various intracranial branches (Figures 3A, 3D). During her hospitalization, she received intravenous codeine and oral acetaminophen. There was partial remission; however, she had two recurrences of sudden headaches associated with vomiting. One of them was associated with post-defecation and the other after the placement of a nasogastric tube. After the last episode, she underwent a brain computed tomography (CT), which showed no new alterations. Subsequently, her clinical evolution was favorable under absolute rest. The computed tomography angiography (CTA) performed 22 days of illness onset showed a partial resolution of the vasoconstriction (Figures 3B, 3E), and at three months a complete resolution (Figures 3C, 3F). The patient had no recurrences until one year of follow-up.

## Discussion

We present the case of a young female who presented with a thunderclap headache associated with focal SAH. Initially, there was suspicion of an aneurysmal etiology; however, the DSA was normal. During her hospitalization, there was a recurrence of thunderclap headache, the MRA showed segmental stenosis of the intracranial arteries, and the diagnosis of RCVS was established. This report highlights the characteristics of thunderclap headache and cSAH as highly guiding findings for RCVS.

RCVS is characterized by a reversible multifocal narrowing of the cerebral arteries associated with recurrent thunderclap headaches with or without focal neurological deficits [1]. Its incidence is unknown, although some series suggest that it is underdiagnosed [2]. In adult occurs between 19 and 70 years of age, (mean 42.5 years) with a predominance in women [2,3], which is consistent with our case. Endogenous or exogenous factors associated with the presence of RCVS may be present in two-thirds [2], among which the delivery, the puerperium, pre-eclampsia/eclampsia, contraceptive drugs, serotonergic drugs, illicit drugs such as cocaine or cannabis, tumors, etc. [2,3]. However, our case corresponds to a spontaneous case.

A thunderclap headache is the most common manifestation of RCVS [2,4-6]. It is defined as a high-intensity headache that peaks in less than 1 minute and mimics the pain of a ruptured cerebral aneurysm [7]. Its accompanying characteristics and the clinical setting make it a very guiding tool for RCVS, even when the initial angiographic study is normal [7]. The first characteristic is that thunderclap headache is usually the initial manifestation. In the series by Singhal et al., thunder headache was the initial symptom in 89% [3], while Ducros et al. reported up to 98.5% [2]. However, our patient started with another type of headache and then did present a thunderclap headache. Another characteristic in RCVS is the recurrence of thunderclap headache, which has a specificity of 99% [8]. In our case, there were five attacks and the last one was at 10 days. The mean described is four to eight attacks between day 1 and day 26 [2,9]. Finally, our patient presented recurrences when performing valsalva maneuvers. This data are important because 71% of thunder headaches are triggered [10] by situations such as sexual intercourse, defecation, sudden emotion, physical effort, effortless urination, coughing, sneezing, bathing or showering, and sudden movements of the head [2]. These three characteristics also allow us to eliminate other diagnostic possibilities that occur concomitantly with vasoconstriction [3].

Another important manifestation of the RCVS is the cSAH. It occurs between 22% and 34% and is usually an early complication, as occurred in our case [2,5]. This isolated convexal pattern would be the result of the rupture of small superficial arteries that undergo dynamic vasodilation-vasoconstriction [2]. In people under 60 years of age, RCVS is considered the main cause of non-traumatic SAH [11]. However, we consider that aneurysmal etiology should always be ruled out since sometimes the visualization of bleeding is not easy or there may be distal aneurysms in unusual locations, although it is rare that they manifest with an isolated convexal pattern [12].

An essential component of diagnosis is to demonstrate cerebral vasoconstriction and its reversibility. Vasoconstriction is usually diffuse, multifocal, and segmental at the level of medium- and large-caliber arteries, alternated with occasional dilated segments that give a similar appearance to a “strip of sausage” [2].

In the pathophysiology of RCVS, sympathetic overactivity, endothelial dysfunction, and oxidative stress [4,13] are implicated in promoting a transitory dysregulation of the tone of the intracranial arteries [4]. The order in the appearance of the symptoms suggests that the affectation of the cerebral tone follows a centripetal gradient. Cortical parenchymal hemorrhages, seizures, posterior reversible leukoencephalopathy, or cSAH usually occur in the first week; while, in the second week, ischemic events of medium and large arteries occur [2]. In addition, as has been discussed, thunder headache is the initial manifestation and generally precedes vasoconstriction [2,9]. This order of presentation was reflected in our case: the first manifestation was headache and then thunder headache; the first imaging finding was cSAH. At that time (seven days sick time) there was no evidence of vasoconstriction in the DSA. This situation was described by Ducros et al., who reported that 21% had normal findings on initial MRA and that it could only be visualized in the second study in a mean of 13.6 days [2]. This is due to the fact that initially there is a compromise of arterioles and leptomeningeal vessels and later the medium and large-caliber vessels are affected. For this reason, in our case, due to clinical suspicion, an angiographic study was repeated on the 15th day of illness, confirming the diagnosis.

The diagnosis of RCVS is based on clinical and neuroimaging characteristics. In our case, the recurrent thunderclap headache, the absence of an aneurysm, and the presence of cSAH established a presumptive diagnosis that was later confirmed by the finding of segmental multifocal vasoconstriction of the cerebral arteries [1,3,8]. The gold standard for the detection of vasoconstriction is DSA; however, for the diagnosis of RCVS, its sensitivity or specificity is not known. In case series studies, non-invasive angiographic studies (CTA and MRA) achieve 70% sensitivity and specificity compared to DSA [14]. A useful tool in the early stages is the RCVS2 score, which distinguishes us between RCVS and other intracranial arteriopathies based on clinical and neuroimaging criteria and does not include the presence of vasoconstriction. Our patient had 10 points on this scale and a score > 5 has a sensitivity and specificity of 90 and 99%, respectively, for RCVS [8]. The benign course described in more than 95% of the patients [15] was reflected in our case, with an almost complete resolution of the vasoconstriction at three months. Recurrence of an episode of RCVS is rare [16].

## Conclusions

Reversible cerebral vasoconstriction syndrome is an increasingly recognized entity. The finding of subarachnoid hemorrhage of the convexity in patients under 60 years of age with no history of trauma should guide us towards this pathology. The time course of its various manifestations, and in our case the initial imaging finding of cSAH, suggests that the vasoconstrictive disorder follows a centripetal course, beginning in small distal arteries and progressing to larger ones. Due to this gradient, the initial studies of blood vessels may not show alterations, making the diagnosis difficult, so it is important that when suspected, these studies are subsequently repeated as was done in the case presented.
